# Increased serum 3-carboxy-4-methyl-5-propyl-2-furanpropanoic acid (CMPF) levels are associated with glucose metabolism in Chinese pregnant women

**DOI:** 10.1007/s40618-017-0789-5

**Published:** 2017-11-18

**Authors:** J. Yi, H. Jin, R. Zhang, S. Zhang, P. Chen, X. Yu, X. Zhang

**Affiliations:** 1 Department of Endocrinology and Metabolism, Jinzhou Medical University, NO. 40 Songpo Road, Section3, Linghe District, Jinzhou, 121001 Liaoning People’s Republic of China; 2 Diabetes Ward, Department of Endocrinology and Metabolism, Fengxian Central Hospital, NO. 6600 Nanfeng Road, Shanghai, 201404 China

**Keywords:** CMPF, Gestational diabetes mellitus, Glucose metabolism, Lipid metabolism, Islet β-cell dysfunction

## Abstract

**Purpose:**

Previous studies have found that 3-carboxy-4-methyl-5-propyl-2-furanpropanoic acid (CMPF) was associated with diabetes. This study aimed to investigate the relationship between abnormal increased CMPF levels and gestational diabetes mellitus (GDM).

**Methods:**

We recruited 828 pregnant women, and all of them underwent an oral glucose tolerance test (OGTT). We screened out 141 GDM patients and 230 pregnant women with normal glucose tolerance as controls. The serum CMPF concentration in participants was measured, and the relationship between the serum CMPF concentration and various parameters and biochemical indices was analyzed.

**Results:**

Compared with the serum levels in pregnant women with normal glucose tolerance, GDM patients exhibited markedly higher serum CMPF levels. The serum CMPF concentration showed an independent positive correlation with the blood glucose levels, glycated hemoglobin A1c(HbA1c), and the area under the glucose–time curve from the 2-h OGTT (AUC for glucose). Moreover, the CMPF concentration was independently negatively correlated with insulin secretion. However, CMPF was not significantly associated with lipid metabolism.

**Conclusions:**

Elevated serum CMPF levels are detrimental to the development of hyperglycemia and islet β-cell functional failure in patients with GDM, which may promote the development of GDM.

**Electronic supplementary material:**

The online version of this article (10.1007/s40618-017-0789-5) contains supplementary material, which is available to authorized users.

## Introduction

Another nationwide epidemiological survey conducted in 2010 showed that the prevalence of diabetes in adults was 11.6% in China [1]. Notably, the prevalence of Gestational diabetes mellitus(GDM) is increasing, GDM and the serious issues caused by GDM cannot be ignored.

The primary risk of uncontrolled GDM is a poor clinical outcome for the mother and the infant [2, 3], and evidence has shown that GDM is a significant risk factor for T2DM. A significant proportion of patients with GDM develop T2DM after childbirth; the rate of developing T2DM within 5-year postpartum was even up to 25–50% [4, 5]. Both GDM and T2DM share the pathological characteristic of islet β-cell function decompensation [6]. However, the etiology of islet β-cell dysfunction is currently unclear. Recently, studies have shown elevated serum CMPF levels in patients with GDM, prediabetes, and T2DM [7, 8], and they infer that CMPF is potentially responsible for promoting the pathogenesis of GDM and the postpartum development of T2DM after GDM.

CMPF is a metabolite produced endogenously from dietary sources of furan fatty acids (FFAs). CMPF also increase upon ingestion of fish, fish oil, and polyunsaturated fatty acids with high-temperature cooking [9]. Small levels of FFAs are also exist in green plants, mushrooms, algae, vegetable oils, and butter, but consumption of these foods was not associated with increased plasma CMPF [10]. Previous studies have demonstrated that CMPF is also a major uremic toxin (UT) [11] and is excreted into the urine via organic anion transporters under physiological conditions [12]. CMPF accumulates at abnormally high levels in the serum of uremic patients due to a decrease of renal clearance [13].

Prentice et al. found a sevenfold upregulation in the levels of CMPF in GDM patients, compared to levels in pregnant women with normal glucose tolerance. In rodent models, Prentice et al. observed dysregulated insulin secretion in CMPF-treated mice. The results indicate that CMPF may be associated with GDM [7]. However, the sample size of their study was limited (only 24 GDM patients and 24 normal glucose tolerance controls). Moreover, the serum CMPF concentrations in the Han Chinese pregnant women remain unknown. In addition, β-cells adaptation to pregnancy may differ between humans and rodents.

Therefore, our objective in the study was to investigate the circulating CMPF levels in a larger cohort of Chinese GDM patients, the association of CMPF with glycolipid metabolism, and the potential link between CMPF and β-cell function.

## Materials and methods

### Study subjects

A total of 828 pregnant women were recruited in this study; all of them were normal glucose tolerant according to the OGTT test prior to pregnancy. They were Han Chinese, and the average age was 28 (25, 30) (years). Subjects with a prior history of diabetes, acute or chronic inflammatory diseases, heart disease, liver disease, kidney disease, or cancer were excluded. Subjects who were taking antihypertensive, hypolipidemic, or hypoglycemic drugs or other medications that were known to affect glucose metabolism during the study period were also excluded. All subjects underwent an OGTT at 24–28 weeks of gestation. We screened out 141 gestational diabetes patients and 230 pregnant women with normal glucose tolerance as controls. The patients and controls were matched by age and pregestational BMI.

### Study materials

A Beckman DXC800 fully automated chemistry analyzer (USA) was used for the measurement of triglycerides, total cholesterol, high-density lipoprotein cholesterol (HDL), low-density lipoprotein cholesterol (LDL), fasting plasma glucose (FPG), OGTT 1-h postprandial glucose (1-h PG), and OGTT 2-h postprandial glucose (2-h PG). A Roche Elecsys 1010 Immunoassay Analyzer and Electrochemiluminescence Immunoassay Kit (Roche Diagnostics, Germany) were used to determine the fasting plasma insulin (FINS), 1-h postprandial insulin (1 h-INS), and 2-h postprandial insulin (2 h-INS). High-pressure liquid chromatography (TOSOH HLC-723 G7, Japan) was used to measure HbA1c.

### Study methods

The height and body weight of the subjects were measured during early gestation. The participants were barefoot and in light clothing, and the pregestational BMI was calculated as the weight divided by height squared (kg/m^2^). At 24–28 weeks of gestation, after an 8-h overnight fast, the subjects had their blood drawn in a quiet state through the antecubital vein, and the fasting blood samples were stored for use later. The subjects were given 75 g of anhydrous glucose through oral administration, and blood samples were collected at 1 and 2 h after glucose administration. The blood samples were centrifuged at 1000 rpm for 15 min to collect the serum, and the biochemical indicators and blood glucose and insulin were measured. Serum samples were stored at − 80 °C until the CMPF concentration measurement was completed.

The diagnostic criteria for GDM were set according to the diagnostic criteria for GDM from the World Health Organization published in 2013, namely, FPG ≥ 5.1 mmol/L, 1-h PG ≥ 10.0 mmol/L, or 2-h PG ≥ 8.5 mmol/L. The homeostatic model assessment index of insulin resistance (HOMA-IR) was calculated as FPG (mmol/L) × FINS (mU/L)/22.5. The homeostatic model assessment index of β-cell secretion (HOMA-β) was calculated as 20 × FINS (mU/L)/(FPG [mmol/L]-3.5). The Stumvoll first-phase and second-phase insulin secretion indices were calculated as 2032 + 4.681 × FINS (pmol/L)-135.0 × 2 h-PG (mmol/L) + 0.995 × 2 h-INS (pmol/L) + 27.99 × BMI (kg/m^2^)-269.1 × FPG (mmol/L) and 277 + 0.800 × FINS (pmol/L)-42.79 × 2 h-PG (mmol/L) + 0.321 × 2 h-INS (pmol/L) + 5.338 × BMI (kg/m^2^), respectively. The area under the glucose–time and insulin–time curve from the 2-h OGTT (AUC for glucose and AUC for insulin, respectively) were calculated as (FPG [mmol/L] + 1 h-PG[mmol/L]) × 1 h/2 + (1 h-PG[mmol/L] + 2 h-PG[mmol/L]) × 1 h/2 and (FINS[mU/L] + 1 h-INS[mU/L]) × 1 h/2 + (1 h-INS[mU/L] + 2 h-INS[mU/L]) × 1 h/2, respectively.

### Measurement of serum CMPF

CMPF levels were measured using an ELISA kit purchased from NOVATEINBIO (USA, batch number: 100902 KB), and the serum samples were not diluted at the time of measurement. The absorbance (OD) values were measured at 450 nm using a microplate reader. The standard curve was generated from the standard samples; the lowest standard concentration in the ELISA kit was 0 ng/mL; and the highest standard concentration was 1000 ng/mL. The CMPF concentration of the serum samples was calculated according to the standard curve.

### Data collection and analysis

Data processing and statistical analyses were performed using the statistical software SPSS19.0. Figures were created using the software GraphPad. The physical parameters and biochemistry indices were compared between the two groups. A normality test was performed for all obtained data; data with a normal distribution were expressed as the mean ± standard deviation, and an intergroup comparison was conducted using a *t* test for two independent samples. The measurement data with a non-normal distribution were expressed using the median (interquartile range), and an intergroup comparison was performed using the Wilcoxon rank-sum test. The parameters with a non-normal distribution underwent logarithmic conversion, and Pearson correlation analysis was used to analyze the correlations between variables. Multivariate linear regression analysis was performed to analyze the influencing factors of serum CMPF. Differences were considered statistically significantly for *p* < 0.05 in two-tailed tests.

## Results

### Physical parameters and biochemical indices of the subjects

Table 1 shows the physical parameters and biochemical indices of the GDM group and the control group with normal glucose tolerance during gestation (NGT group).

**Table 1:** Clinical characteristics of the NGT and GDM participants

Variable	NGT (*n* = 230)	GDM (*n* = 141)	*p* value
Age (year)	29 (25, 32)	29 (26, 32.50)	0.529
Pregestational body mass index(kg/m^2^)	21.604 (19.835, 23.438)	22.039 (19.988, 23.673)	0.271
Systolic blood pressure (mmHg)	110 (104, 120)	112 (102, 120)	0.267
Diastolic blood pressure (mmHg)	70 (65, 75)	70 (65.50, 78)	0.353
Alkaline phosphatase (U/L)	58 (48, 69.25)	59.5 (49, 70)	0.321
Alanine aminotransferase (U/L)	14 (10, 22)	13 (10, 21.50)	0.651
Aspartate aminotransferase (U/L)	18.50 (16, 23)	19 (15, 24)	0.840
Creatinine (μmol/L)	46.98 ± 6.18	46.67 ± 6.66	0.631
eGFR (mL/min 1.73 m^2^)	148.87 (135.42, 170.03)	153.45 (133.89, 169.01)	0.832
Hemoglobin A1C (%)	4.71 (4.50, 4.90)	4.90 (4.60, 5.20)	0.001**
FPG (mmol/L)	4.30 (4.00, 4.50)	4.90 (4.40, 5.90)	< 0.001**
1 h-PG (mmol/L)	6.60 (5.70, 7.80)	9.50 (7.60, 10.90)	< 0.001**
2 h-PG (mmol/L)	6.05 (5.30, 6.80)	8.50 (6.10, 9.20)	< 0.001**
AUC for glucose (mmol/L h)	11.88 ± 1.83	15.85 ± 2.93	< 0.001**
Triglycerides (mmol/L)	2.27 (1.79, 2.91)	2.39 (1.91, 2.97)	0.241
Total cholesterol (mmol/L)	6.17 (5.64, 6.92)	6.00 (5.27, 6.93)	0.097
HDL-c (mmol/L)	1.67 (1.53, 1.88)	1.67 ± 0.29	0.269
LDL-c (mmol/L)	2.91 ± 0.74	3.00 ± 0.88	0.569
FINS (mU/L)	8.68 (5.89, 12.94)	10.26 (6.63, 15.46)	0.011*
1 h-INS (mU/L)	62.67 (43.77, 89.78)	74.23 (48.65, 117.00)	0.020*
2 h-INS (mU/L)	60.74 (40.45, 84.73)	83.925 (52.01, 134.00)	< 0.001**
AUC for insulin (mU/L h)	100.45 (70.13, 136.29)	126.81 (84.55, 184.60)	0.001**
HOMA-β	248 (173.50, 367)	169.10 (93.39, 255.72)	< 0.001**
HOMA-IR	1.65 (1.10, 2.49)	2.24 (1.45, 3.84)	< 0.001**
Stumvoll 1st phase insulin secretion index	1393.88 (1243.77, 1714.35)	1176.93 (863.62, 1660.09)	0.001**
Stumvoll 2nd phase insulin secretion index	173.09 (139.07, 194.36)	80.79 (33.51, 141.23)	0.067

Values are presented as the mean ± SD or medians (interquartile range)

*eGFR* estimated glomerular filtration rate, *FPG* fasting plasma glucose, *1* *h-PG* 1-h postprandial glucose, *2* *h-PG* 2-h postprandial glucose, *AUC for glucose* area under the glucose–time curve from the 2-h OGTT, *HDL* high-density lipoprotein cholesterol, *LDL-c* low-density lipoprotein cholesterol, *FINS* fasting insulin, *1* *h-INS* 1-h postprandial insulin, *2* *h-INS* 2-h postprandial insulin, *AUC for insulin* area under the insulin–time curve from the 2-h OGTT, *HOMA-β* homeostasis model assessment index of β-cell secretion, *HOMA-IR* homeostasis model assessment of insulin resistance

*Significantly different compared to NGT at *p* < 0.05

**Significantly different compared to NGT at *p* < 0.01

Statistically significant differences were found between the two groups in FPG, 1 h-PG, 2 h-PG, AUC for glucose, HbA1c, FINS, 1 h-INS, 2 h-INS, AUC for insulin, HOMA-IR and HOMA-β, and the Stumvoll first-phase insulin secretion index. Compared to the levels in the NGT control group, the GDM group exhibited a higher FPG, 1 h-PG, 2 h-PG, AUC for glucose, HbA1c, FINS, 1 h-INS, 2 h-INS, AUC for insulin, and HOMA-IR. The NGT group had a higher HOMA-β and the Stumvoll first-phase insulin secretion index than the GDM group.

**Table 2:** Multiple stepwise linear regression analysis of the variables independently associated with serum CMPF levels

	*Β*	SE	Standardized *β*	*p*
Stumvoll 1st phase insulin secretion index (kg/m^2^)^a^	− 0.181	0.089	− 0.141	0.043

The model is adjusted for age, Pre-pregnant BMI, triglycerides, HDL, HOMA-IR, and HbA1C

^a^Log-transformed

### Elevated serum CMPF levels in subjects with GDM

**Fig. 1:**
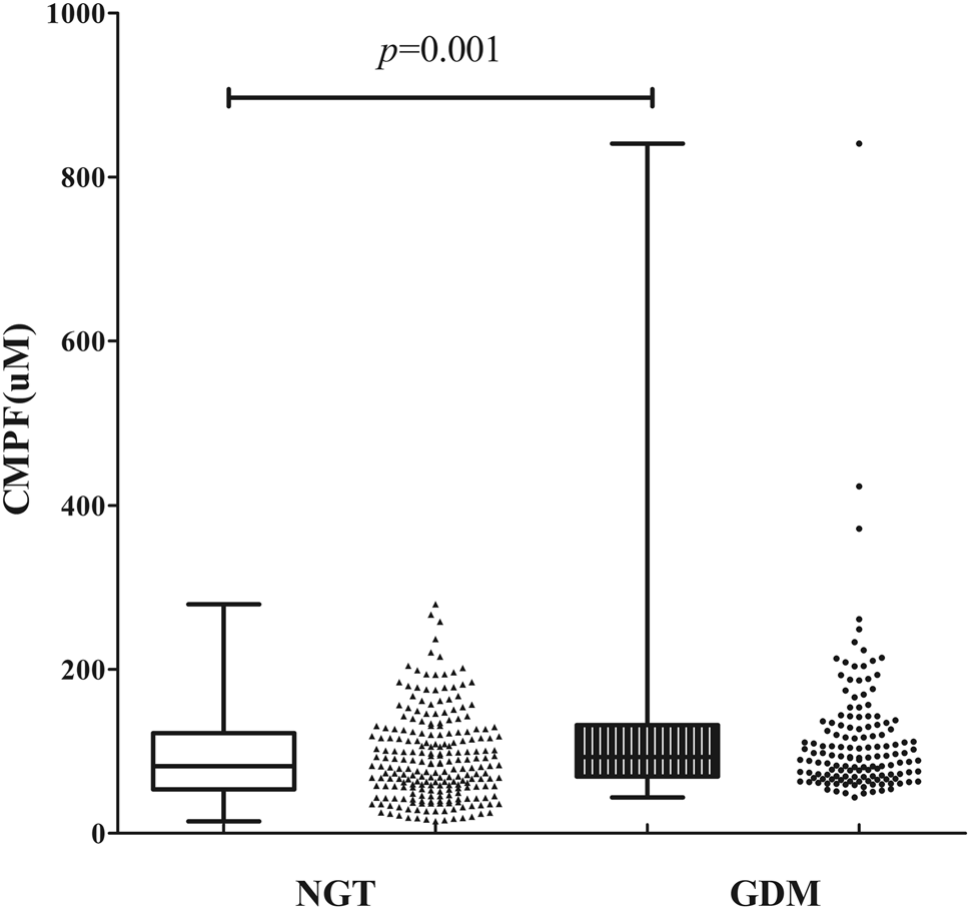
Plasma CMPF is Elevated in Gestational Diabetes Mellitus Populations. The plasma CMPF level was evaluated in fasting plasma samples of pregnant women collected in 2015–2016; *n* = 230 and 141 for the gestational diabetes and normal glucose tolerance groups, respectively. The two groups were matched based on age and pregestational BMI. Values are presented in scatter plot, and medians (interquartile range) are expressed in box plot. *P* value is statistically significant at *p* < 0.05

Compared to the level in the NGT group, the GDM group had a significantly higher serum CMPF level. The CMPF concentration was 92.88 (69.42, 132.13) (μM) and 81.95 (53.68, 122.33) (μM) in the GDM group and the NGT group, respectively (*p* = 0.001). The comparison of the CMPF concentration between these two groups is shown in Fig. 1.

### Relationship between CMPF and glucose metabolism

**Fig. 2:**
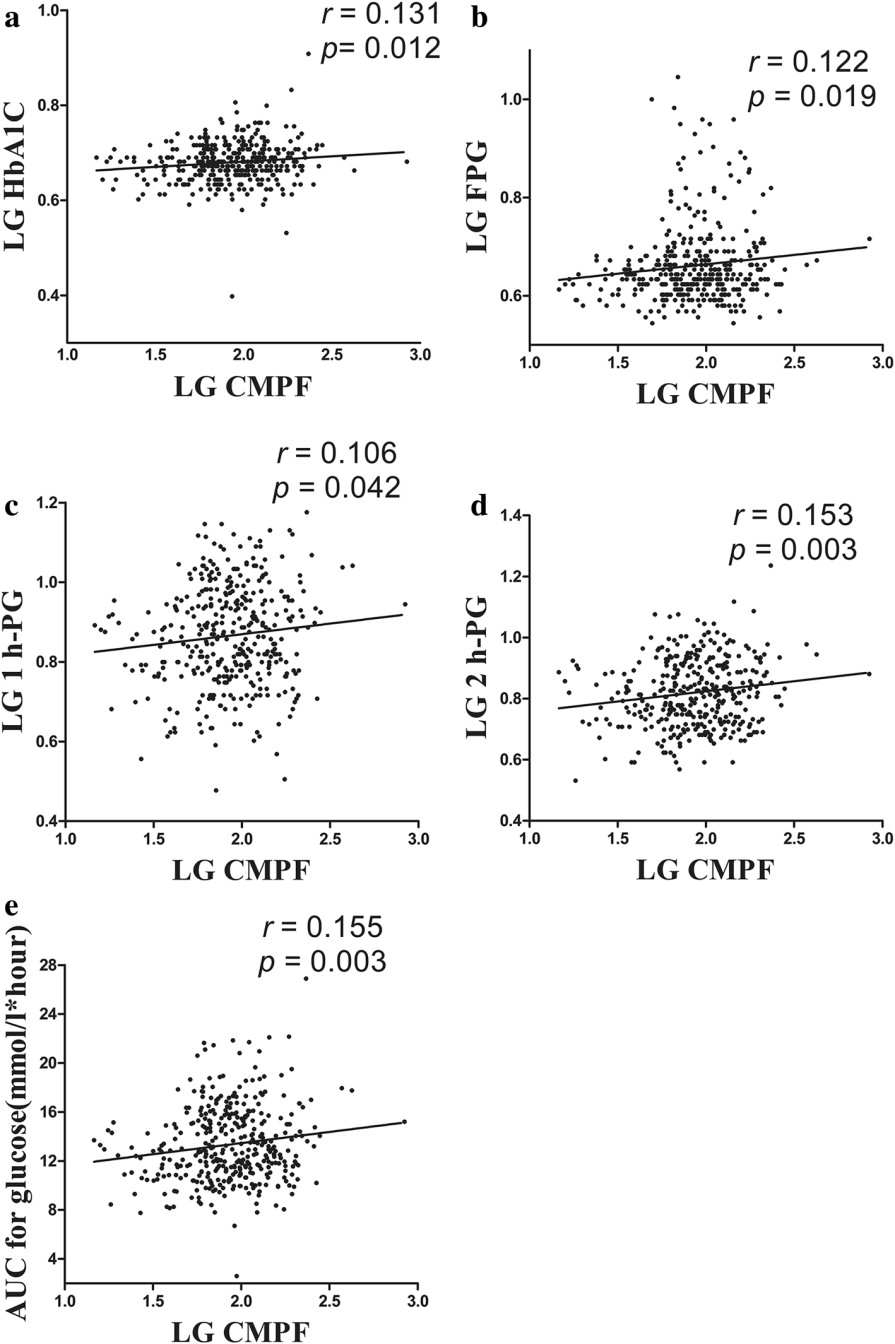
Elevated CMPF impairs glucose metabolism in gestational diabetes mellitus populations. **a**–**e** Correlation analyses between CMPF and HbA1c, FPG, 1 h-PG, 2 h-PG, and AUC for glucose, respectively. Pearson’s bivariate correlation analyses showed that CMPF was positively correlated with HbA1c, FPG, 1 h-PG, 2 h-PG, and AUC for glucose. A 2-h OGTT was performed, and the corresponding blood glucose levels and HbA1c were determined in the gestational diabetes and normal glucose tolerance groups. AUC for glucose: *n* = 140 for GDM and *n* = 225 for NGT. *LG* log-transformed

A bivariate correlation analysis was conducted with the CMPF levels and variables related to glucose metabolism. CMPF, HbA1c, FPG, 1 h-PG, and 2 h-PG showed a non-normal distribution, while the AUC for glucose showed a normal distribution. After logarithmic conversion of the variables with a non-normal distribution, CMPF was found to exhibit an independent positive correlation with HbA1c (*r* = 0.131, *p* = 0.012), FPG (*r* = 0.122, *p* = 0.019), 1 h-PG (*r* = 0.106, *p* = 0.042), 2 h-PG (*r* = 0.153, *p* = 0.003), and AUC for glucose (*r* = 0.155, *p* = 0.003). In Fig. 2, panels a, b, c, d, and e represent the bivariate correlation between CMPF and HbA1c, FPG, 1 h-PG, 2 h-PG, and AUC for glucose, respectively. After an adjustment for age and pregestational BMI, a partial correlation analysis indicated an independent positive correlation of CMPF with HbA1c (*r* = 0.111, *p* = 0.041), FPG (*r* = 0.118, *p* = 0.027), 1 h-PG (*r* = 0.106, *p* = 0.048), 2 h-PG (*r* = 0.147, *p* = 0.006), and AUC for glucose (*r* = 0.143, *p* = 0.007).

### Serum CMPF and lipid metabolism

We examined whether CMPF had a different effect on lipid metabolism in pregnant women. CMPF, triglycerides, total cholesterol, HDL, and LDL showed a non-normal distribution. After logarithmic conversion, the bivariate correlation analysis with CMPF and variables related to lipid metabolism showed that CMPF had no apparent correlation with triglycerides (*r* = − 0.047, *p* = 0.374), total cholesterol (*r* = − 0.018, *p* = 0.727), HDL (*r* = 0.009, *p* = 0.864), or LDL (*r* = 0.042, *p* = 0.412). After an adjustment for age and pregestational BMI, similar results were obtained from the partial correlation analysis, and no significant correlation was observed between serum CMPF and triglycerides (*r* = − 0.072, *p* = 0.179), total cholesterol (*r* = − 0.015, *p* = 0.777), HDL (*r* = 0.022, *p* = 0.683), or LDL (*r* = 0.055, *p* = 0.304).

### CMPF and islet β-cell dysfunction

**Fig. 3:**
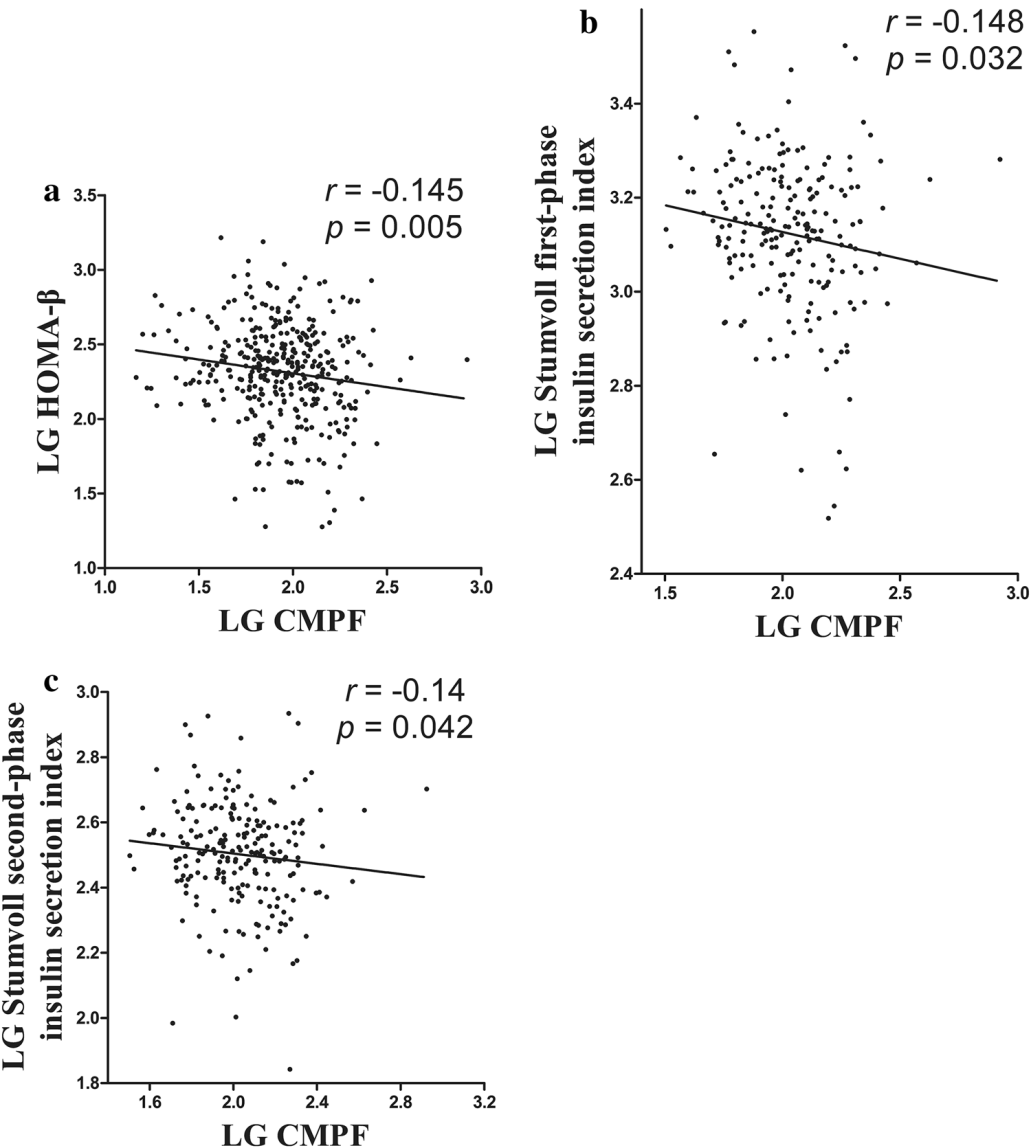
Elevated CMPF impairs glucose-stimulated insulin secretion. **a**–**c** Correlation analysis between CMPF and HOMA-β, the Stumvoll first-phase insulin secretion index, and the Stumvoll second-phase insulin secretion index values, respectively. A 2-h OGTT was performed, and the corresponding blood insulin levels were examined in the gestational diabetes and normal glucose tolerance groups, respectively. AUC for insulin: *n* = 86 for GDM and *n* = 130 for NGT. *LG* log-transformed

CMPF, HOMA-β, HOMA-IR, and the Stumvoll first-phase and second-phase insulin secretion indexes showed a non-normal distribution. After logarithmic conversion, a bivariate correlation analysis showed an independent negative correlation between CMPF and HOMA-β (*r* = − 0.145, *p* = 0.005) and no statistically significant correlation between CMPF and HOMA-IR (*r* = 0.002, *p* = 0.962). Furthermore, CMPF showed an independent negative correlation with the Stumvoll first-phase insulin secretion index (*r* = − 0.148, *p* = 0.032) and the Stumvoll second-phase insulin secretion index (*r* = − 0.14, *p* = 0.042). After an adjustment for age and pregestational BMI, the partial correlation analysis showed that CMPF had an independent negative correlation with HOMA-β (*r* = − 0.134, *p* = 0.012) and the Stumvoll first-phase insulin secretion index after logarithmic conversion (*r* = − 0.138, *p* = 0.046). In Fig. 3, panels a, b, and c show the bivariate correlation between serum CMPF and HOMA-β, the Stumvoll first-phase insulin secretion index, and the Stumvoll second-phase insulin secretion index, respectively.

A multivariate linear regression analysis showed that serum CMPF was negatively correlated with the Stumvoll first-phase insulin secretion index (*β* = − 0.181 ± 0.089, *p* = 0.043) after an adjustment for age, pregestational BMI, triglycerides, HDL, HOMA-IR, and HbA1c (Table 2).

## Discussion

The serum CMPF concentration in pregnant women was measured under two different glucose metabolism states, and the concentration of CMPF in subjects with GDM was higher than in pregnant women with normal glucose tolerance. Notably, women with GDM do not usually have renal failure, and the reason for this difference of CMPF levels in GDM and controls is not clear. In addition, we analyzed the results and found that the CMPF concentration was closely related to glucose metabolism and islet β-cell dysfunction.

Previous studies have given different conclusions regarding the role of CMPF in the pathological process of diabetes. Prentice et al. found upregulated levels of CMPF in patients with GDM, and in a subset of the same women who had GDM and became impaired glucose tolerant, CMPF was even more dramatically elevated 12-fold 1-year postpartum compared to its levels in women who were normal glucose tolerance at both timepoints. Moreover, CMPF impairs glucose tolerance and glucose utilization in rodent models [7]. The study of Liu et al. showed that CMPF is significantly elevated in both prediabetes and T2DM, and individuals with an increase in plasma CMPF concentrations were at a significantly higher risk of developing diabetes within 5 years. They also found that mice treated with CMPF exhibited an even greater impairment in glucose tolerance than control mice [8]. Our results are in accordance with the findings from the studies by Prentice and Liu et al., which supports the speculation that CMPF is inseparable from the disruption of the glucose metabolism balance.

However, in a double-blind, randomized controlled study in China, Zheng et al. found that the serum CMPF levels in 59 Chinese patients with T2DM were lower than those in healthy control subjects [14]. Lankinen et al. found in their diet intervention study that an elevated serum CMPF level was not significantly associated with impaired glucose metabolism [15]. In the study by Retnakaran et al., 307 postpartum women underwent an OGTT test, and 66 subjects were found to have diabetes or prediabetes, while 301 subjects showed normal glucose tolerance; however, no significant differences were observed in the serum CMPF concentrations between the two groups [16]. These results are opposite to our findings. Age, race, dietary habits, study methods, and the diabetes duration might influence serum CMPF levels and cause these differences. The relationship between CMPF and glucose metabolism requires further verification using a larger sample size.

Lipids are an important risk factor for diabetes and cardiovascular diseases; therefore, the previous studies have examined whether CMPF affects lipid metabolism. Zheng et al. found an inverse correlation between changes in CMPF and triglycerides [14]. However, no obvious relationship was observed between CMPF and lipids in our study. Whether CMPF affects lipid metabolism awaits further study.

Previous studies have also provided different opinions regarding the relationship between CMPF and islet β-cell dysfunction. Lankinen et al. found in their diet intervention study that an elevated serum CMPF level was correlated with reduced 2 h-INS secretion in the OGTT test [15]. In the study by Retnakaran et al., the serum CMPF levels did not exhibit an apparent correlation with postpartum insulin sensitivity, insulin resistance, or islet β-cell function. However, their subsequent study showed that in patients with GDM, high levels of CMPF were independently correlated with poor islet β-cell function, while this correlation was not found in women with normal glucose tolerance. Retnakaran et al. proposed that CMPF could be a potential determinant of islet β-cell dysfunction in GDM [16].

Moreover, in rodent models, Prentice et al. found that elevated CMPF impairs glucose-stimulated insulin secretion, increases advanced glycation endproducts and oxidative stress, impairs insulin granule maturation, and accelerates the process of diabetes. Mechanistically, Prentice et al. showed that CMPF acts directly on the β-cell, causing impaired mitochondrial function, decreasing glucose-induced ATP accumulation, and inducing oxidative stress, resulting in dysregulation of key transcription factors and ultimately reduced insulin biosynthesis [7]. Liu et al. also observed that CMPF treatment impairs insulin granule maturation in mice [8]. These results observed in their experiments are consistent with the conclusion from our study.

However, due to the limited sample size in our study and the contrary conclusions reported in other studies, the conclusions await further study for confirmation. The main limitation of our study is that it was a cross-sectional study and did not reflect the effect of CMPF on glucose metabolism and islet β-cell function over time. Second, only the fasting serum CMPF concentration was measured in the present study, and the postprandial serum CMPF concentration was not examined. Third, frozen serum rather than fresh serum was used in this study. There were no differences in CMPF levels between frozen and fresh samples. In addition, no adjustment of multiple testing was performed on the analyses of circulating CMPF concentration and clinical parameters. Given the modest *p* values, most of the tests would not be significant with a Bonferroni correction. However, Bonferroni correction is not appropriate here as the parameters were closely related, not independent, adjustment for multiple comparisons with Bonferroni correction is too strict.

In summary, we infer that CMPF may be an important substance in the development of hyperglycemia and islet β-cell functional failure in patients with GDM and an important moderator associated with postpartum T2DM. Therefore, future studies will be critical for understanding the relationship between CMPF and GDM and T2DM, and studies of CMPF should focus on the effect of CMPF in different tissues and organs of the human body.

## Electronic supplementary material

Below is the link to the electronic supplementary material.

Supplementary material 1 (DOC 11 kb)

Supplementary material 2 (JPEG 6542 kb)
