# Supplementary material for: Increased serum 3-carboxy-4-methyl-5-propyl-2-furanpropanoic acid (CMPF) levels are associated with glucose metabolism in Chinese pregnant women
Source: J Endocrinol Invest. 2017 Nov 18;41(6):663–70. doi: 10.1007/s40618-017-0789-5 (PMC5951875; doi:10.1007/s40618-017-0789-5)
Supplement: Supplementary file 1 — Supplementary material 1 (DOC 11 kb) [file 40618_2017_789_MOESM1_ESM.doc]

Data Availability Statement:

Due to the sensitive nature of the data and the consent agreements signed by participants, data cannot be made publicly available. Data are available upon request. Please include a proposal for use of the data, which will be submitted to the authors' ethics committee for approval. Requests for the data may be sent to xuemeiyu12@163.com. The authors confirm that all interested parties will be able to obtain the data via the contact provided after some documentation is completed.
