# Supplementary figures and images for: Increased serum 3-carboxy-4-methyl-5-propyl-2-furanpropanoic acid (CMPF) levels are associated with glucose metabolism in Chinese pregnant women
Source: J Endocrinol Invest. 2017 Nov 18;41(6):663–70. doi: 10.1007/s40618-017-0789-5 (PMC5951875; doi:10.1007/s40618-017-0789-5)

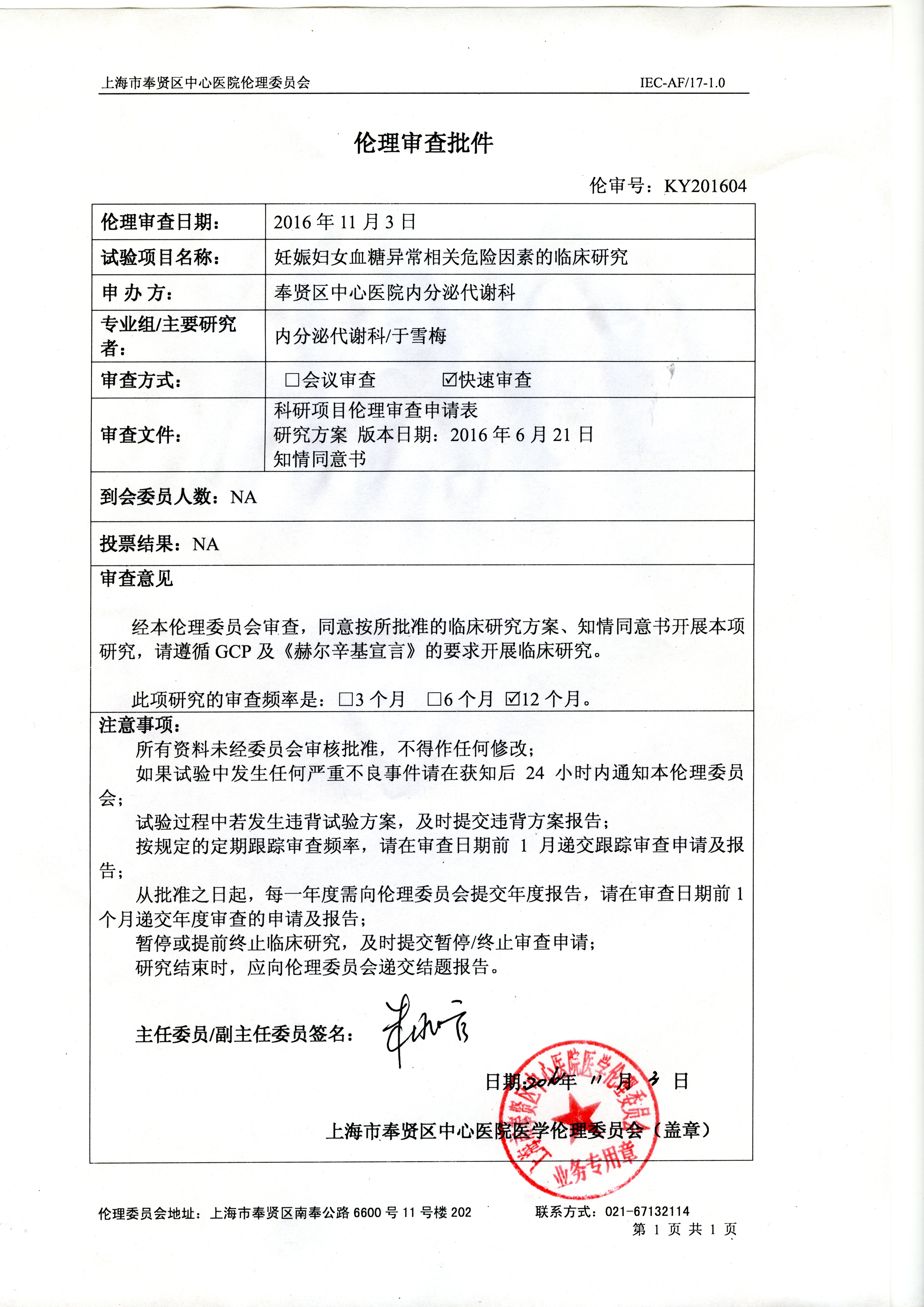

Supplement: Supplementary file 2 — Supplementary material 2 (JPEG 6542 kb) [file 40618_2017_789_MOESM2_ESM.jpg]
